# Pulmonary Langerhans Cell Histiocytosis Presenting as a Solitary Pulmonary Nodule on a Lung Cancer Screening CT

**DOI:** 10.1155/2020/8872111

**Published:** 2020-12-28

**Authors:** Hassan Baig, Sushilkumar Sonavane, Ian Makey, Jorge Mallea, Andras Khoor

**Affiliations:** ^1^Department of Pulmonary and Critical Care Medicine, Mayo Clinic Florida, 4500 San Pablo Road, Jacksonville, FL 32224, USA; ^2^Department of Diagnostic Radiology, Mayo Clinic Florida, 4500 San Pablo Road, Jacksonville, FL 32224, USA; ^3^Department of Thoracic Surgery, Mayo Clinic Florida, 4500 San Pablo Road, Jacksonville, FL 32224, USA; ^4^Department of Anatomic and Clinical Pathology, Mayo Clinic Florida, 4500 San Pablo Road, Jacksonville, FL 32224, USA

## Abstract

Pulmonary Langerhans cell histiocytosis (PLCH) is a rare inflammatory condition that mostly affects lungs in smokers. On imaging, it usually presents as multiple, upper lobe predominant, solid, and cavitary nodules, but presentation as solitary pulmonary nodule (SPN) is rare. We describe a case of SPN seen on low-dose lung cancer screening CT (LDCT) that was FDG avid on PET/CT. Given concern for malignancy, lobectomy was planned if intraoperative frozen section was consistent with malignancy. Lobectomy was performed based on frozen section; however, on formal pathology review, the nodule was ultimately found to be PLCH. This case illustrates an atypical presentation of PLCH as a solitary nodule. Furthermore, it helps demonstrate how rare etiologies (like PLCH) may be more frequently encountered and should be considered in the differential diagnosis for solitary lung nodules, especially in the era of lung cancer screening.

## 1. Background

The evaluation of a solitary pulmonary nodule (SPN) involves appropriate risk stratification for malignancy and weighing potential harms of interventions, especially in the current era of computed tomography (CT) screening for lung cancer. The etiology of lung nodules seen in lung cancer screening programs is predominantly benign [1] such as from infection, inflammation, or granulomatous disease [2]. Pulmonary Langerhans cell histiocytosis (PLCH) is a rare inflammatory condition that mostly affects lungs in smokers [3]. On imaging, it can be seen as multiple, upper lobe predominant, solid, and cavitary nodules, but presentation as a solitary pulmonary nodule is rare. We describe a case of a SPN seen on low-dose lung cancer screening CT (LDCT) that was suspicious on positron emission tomography (PET) scan and was ultimately found to be PLCH on surgical pathology. This case presented a diagnostic dilemma, as intraoperative frozen sections were suggestive of malignancy.

## 2. Case Presentation

**Figure 1:**
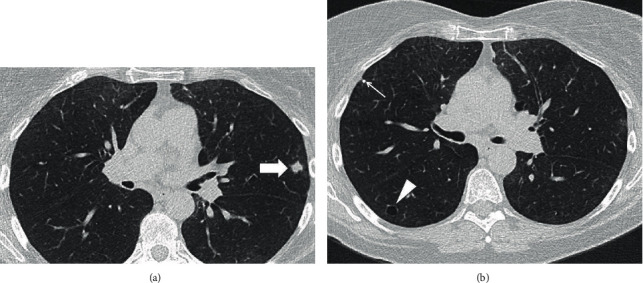
(a, b) Axial chest CT images show an irregular shape, 1.1 cm, left upper lobe nodule with spiculated margins (block arrow), and a thin-walled parenchymal cyst in right lower lobe (arrowhead). Please note mild centrilobular, paraseptal emphysema, and a tiny, calcified right upper lobe nodule (thin arrow).

**Figure 2:**
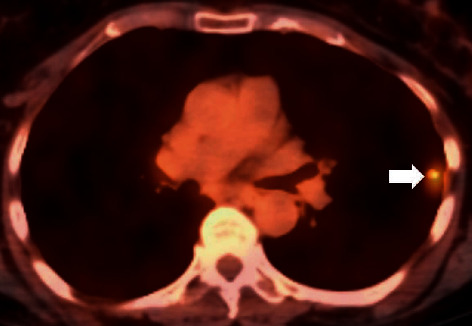
Axial, fused FDG PET–CT image shows increased FDG uptake in the left upper lobe nodule with SUV of 4.2 (arrow).

A 63-year-old female patient, current smoker with a 15 pack-year history, presented to the pulmonary clinic for evaluation and management of a single lung nodule detected on LDCT at another facility. The LDCT chest demonstrated a left upper lobe, 11 mm nodule with spiculated borders. Other imaging features included upper lobe predominant centrilobular emphysema, a few, tiny, calcified nodules, presumably old granulomas, and two small, thin-walled parenchymal cysts (Figure 1). She had already completed a whole body PET/CT scan as recommended. The PET/CT scan displayed (18)F-fluorodeoxyglucose (FDG) avidity of the nodule with a standardized uptake value (SUV) of 4.2 (Figure 2) and no abnormal uptake elsewhere in the body. Her symptoms included occasional nonproductive cough. She denied significant shortness of breath, fever, chills, night sweats, weight loss, or hemoptysis.

Given the patient's age, smoking status, and imaging characteristics, the concern for lung cancer was high. After evaluation by the thoracic surgery team and presentation at a multidisciplinary conference, it was decided to proceed with diagnostic wedge resection and subsequent left upper lobectomy if indicated. During the procedure, frozen section analysis of the wedge resection showed a 10 mm nodule with atypical alveolar type II cell proliferation. Adenocarcinoma with lepidic features was suspected, and the left upper lobectomy was completed. The patient was discharged without complications.

**Figure 3:**
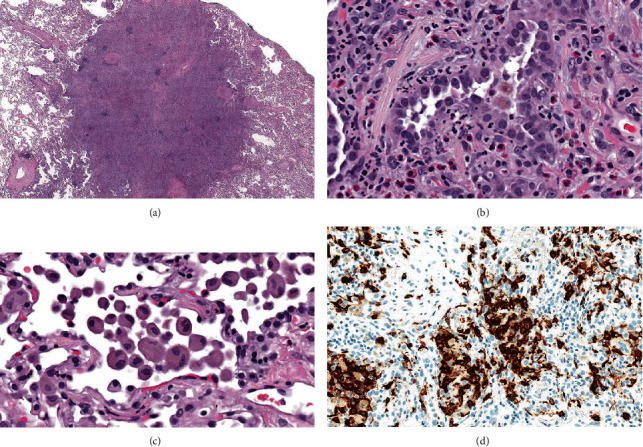
Histologic features of the pulmonary nodule. (a) Low magnification image shows a solid appearing nodule with indistinct edges (hematoxylin-eosin stain, original magnification 8x). (b) A high magnification view exhibits marked reactive alveolar type II cell hyperplasia. The alveolar septa are expanded and contain scattered eosinophils (hematoxylin-eosin stain, original magnification 400x). (c) Another high magnification view shows alveolar macrophage accumulation at the edge of the nodule (hematoxylin-eosin stain, original magnification 400x). (d) Immunohistochemistry for langerin reveals clusters of Langerhans cells within the nodule (original magnification 400x).

Sections of the formalin-fixed paraffin-embedded nodule revealed alveolar septal expansion with numerous eosinophils, alveolar macrophage accumulation (desquamative interstitial pneumonia-like reaction), and focal organizing pneumonia (Figure 3). The alveolar type II cell proliferation, which had appeared atypical in the previous frozen sections, now looked reactive in nature. The histologic findings were consistent with pulmonary Langerhans cell histiocytosis. Immunohistochemical studies exhibited clusters of S100, CD1a, and langerin-positive Langerhans cells within the lesion, confirming the diagnosis.

In the outpatient setting, the patient was advised smoking cessation as the most important intervention to prevent disease progression. Regular follow-up every 3-6 months initially was advised with pulmonary function tests and chest CT to assess for further lung parenchymal involvement in the form of worsening nodular or cystic disease and the development of fibrosis.

## 3. Discussion

Lung nodules are commonly detected incidentally on chest radiographs, routine chest CT, or LDCT for lung cancer screening. The overall chance of the nodule being cancerous, even among high-risk patients, tends to be low [1]. Certain imaging features such as nodule size and spiculated margins can raise the suspicion for malignancy. PET/CT scan provides useful information for lung cancer diagnosis and staging but can have a false-positive rate of 25% and even up to 40% in areas with endemic infections such as fungal or tuberculous [4]. In our case, the patient's smoking status, nodule size of 11 mm, and high FDG avidity on PET/CT scan led to further surgical diagnostic evaluation. Of note, the calculated lung cancer risk based on a validated tool (Brock University cancer prediction equation) was 31% [1], and 80% was accounting for the PET/CT data (based on Herder prediction model) [5], conferring a moderate to high malignant risk. Due to a possibility of a nonnegligible false-negative result, CT-guided biopsy might not have been sufficient to confidently rule out malignancy. Nondiagnostic biopsies in lesions less than 3 centimeters have been reported in up to 36% of cases [6]. This and other aspects of the different diagnostic interventions were discussed with patient. She actively participated in the shared decision-making process, highlighting the importance of such a process when ordering and discussing the results of screening CTs.

After formal pathology review, the diagnosis of pulmonary Langerhans cell histiocytosis was made. The initial diagnostic wedge resection demonstrated a nodule with significant atypical type II alveolar cell proliferation. This raised the suspicion of adenocarcinoma; however, on formal pathology review after lobectomy, it was not the case. Small lung adenocarcinoma diagnosis can pose challenges on frozen section because alveolar cell atypia and artifacts can simulate malignancy. Frozen section analysis can have an error rate up to 6% [7]. Furthermore, a sublobar resection instead of lobectomy could have been considered. Based on the only randomized controlled trial, limited resections are associated with higher local recurrent rates, lower overall survival, and equivalent perioperative morbidity as compared to lobectomy [8]. However, increasing evidence from observational studies has demonstrated similar outcomes including recurrence rates and overall survival in those patients intentionally chosen to undergo sublobar resection (as opposed to necessity due to underlying comorbidities) [ 9]. Further, randomized trials are underway, and until then, the standard of care in a low-surgical-risk patient remains lobectomy.

PLCH is an interstitial lung disease closely linked to smoking. Clinically, it is characterized by shortness of breath and cough with minimal crackles on lung auscultation [3]. On imaging, the upper and mid lung zones show greater involvement compared to lower lung zones. Small, stellate, or irregular-shape centrilobular nodules are present, some of which can show faint central lucencies. The nodules are usually solid density and can also have surrounding ground glass density [10]. In advanced stage of the disease, the cysts become larger, irregular, or bizarre shaped [11]. Histologically, the disease is characterized by peribronchiolar nodules, containing a mix of smoker's macrophages, eosinophils, and Langerhans cells along with varying degrees of fibrosis. Langerhans cells can be identified by their pale blue nucleus with nucleolar infoldings and with special stains including S100, CD1a, and langerin [12].

Management of PLCH typically involves smoking cessation with clinical and imaging follow-up as necessary. If the disease progresses, steroids and even chemotherapy can be considered [13]. Lung transplantation is reserved for severe, intractable cases [14].

The unique aspect of this case is that PLCH presenting as a solitary pulmonary nodule is extremely rare. Only a handful of cases have been described that presented similarly as a SPN concerning for malignancy and were resected [15–17]. On follow-up imaging ranging from 2 up to 24 years, no significant sequelae had been noted in these reported cases. Furthermore, in the era of low-dose CT scans for lung cancer screening, nodules requiring further diagnostic workup are likely to become more prevalent and will require careful consideration of all etiologies, including those less commonly encountered.

## Data Availability

Further details and information about the case are available upon request. The contact person is Hassan Baig (baig.hassan@mayo.edu).
